# Intact high‐resolution working memory binding in a patient with developmental amnesia and selective hippocampal damage

**DOI:** 10.1002/hipo.23452

**Published:** 2022-06-23

**Authors:** Richard J. Allen, Amy L. Atkinson, Faraneh Vargha‐Khadem, Alan D. Baddeley

**Affiliations:** ^1^ School of Psychology University of Leeds Leeds UK; ^2^ Department of Psychology Lancaster University Lancaster UK; ^3^ Developmental Neurosciences Department University College London Great Ormand Street Institute of Child Health London UK; ^4^ Department of Psychology University of York York UK

**Keywords:** developmental amnesia, hippocampus, memory precision, short‐term memory, visual working memory

## Abstract

Debate continues regarding the possible role of the hippocampus across short‐term and working memory tasks. The current study examined the possibility of a hippocampal contribution to precise, high‐resolution cognition and conjunctive memory. We administered visual working memory tasks featuring a continuous response component to a well‐established developmental amnesic patient with relatively selective bilateral hippocampal damage (Jon) and healthy controls. The patient was able to produce highly accurate response judgments regarding conjunctions of color and orientation or color and location, using simultaneous or sequential presentation of stimuli, with no evidence of any impairment in working memory binding, categorical accuracy, or continuous precision. These findings indicate that hippocampal damage does not necessarily lead to deficits in high‐resolution cognitive performance, even when the damage is severe and bilateral.

## 1 INTRODUCTION

A long‐standing distinction has been drawn between short‐term or working memory (WM) and long‐term memory. Much of the evidence for such a distinction has been provided by neuropsychological case studies, showing impaired long‐term memory (LTM) alongside preserved short‐term memory (STM; e.g., Baddeley & Warrington, 1970; Milner, 1966) or vice versa (Shallice & Warrington, 1970; Vallar & Baddeley, 1984). However, debate continues concerning the relationship between temporary and long‐term retention. This debate has been focused at the theoretical level but has heavily drawn on the neuropsychological literature (see, e.g., Baddeley et al., 2019, 2021; Buchsbaum & D'Esposito, 2019; Cowan, 1988; Hanley & Young, 2019; Logie, 2019; Morey, 2018; Morey et al., 2019; Shallice & Papagno, 2019).

One issue that is relevant to this debate is the extent to which the hippocampus and broader medial temporal lobe (MTL) structures are involved in both WM and LTM, or only the latter (see Allen (2018) for a brief review). The more established view is for a separation, with the hippocampus playing an important role in key forms of cognition such as episodic memory, spatial awareness, and navigation, while not critically contributing to working memory (e.g., Eichenbaum, 2006; Eichenbaum et al., 1994). An alternative possibility is that the hippocampus may contribute to any memory task, regardless of load or duration, particularly if it is recall‐based. For example, substantial evidence has implicated an important role for the hippocampus in episodic LTM, particularly regarding the way in which disparate elements of an episode are associated or bound together to form a coherent representation (e.g., Davachi, 2006; Eichenbaum et al., 2007). Building on this, it has been argued that binding in working memory also has a key hippocampal or MTL component, with studies reporting deficits in patients with hippocampal and/or broader MTL damage in tasks designed to measure binding and/or associative memory over the short term (e.g., Hannula et al., 2015; Olson et al., 2006; Pertzov et al., 2013; Zokaei et al., 2019). We would note in this context though that it is difficult to attribute reported deficits specifically to hippocampal or parahippocampal function, given that these studies often report on adult‐acquired amnesia resulting from hippocampal plus broader MTL damage. Furthermore, adult‐onset injury hippocampal injury with or without MTL involvement usually occurs in previously healthy people who had relied on a normally organized memory circuit with access to both working memory and LTM operating in tandem. A very different pattern of hippocampal/MTL interaction can result when the injury occurs before the memory circuit has functionally developed, with plastic changes in the MTL cortices potentially altering the balance between working memory and LTM contributions during memory formation (Elward & Vargha‐Khadem, 2018).

Indeed, it has been suggested that apparent evidence for hippocampal involvement in WM in fact reflects LTM contribution to task performance (Jeneson et al., 2010, 2012; Jeneson & Squire, 2012; Shrager et al., 2008). Thus, a task ostensibly designed to index WM might exceed WM capacity or temporal duration, forcing the participant to draw on LTM to supplement performance, thus increasing the chance of observing an apparent deficit in patients. This illustrates the importance of careful task design and the principle noted by Atkinson and Shiffrin (1968) (see Baddeley et al., 2019) that few experimental tasks are process pure and that both short‐term and long‐term storage components are likely to be simultaneously active in experiments designed to capture STM. In line with this, case study work with Jon, a patient with selective hippocampal damage, has repeatedly shown intact (and indeed often somewhat superior) performance on a range of tasks carefully designed to focus on working memory including measures of binding between shape, color, or location (Allen et al., 2014; Baddeley et al., 2010, 2011), alongside severely impaired episodic LTM abilities.

One related possibility is that the hippocampus contributes to performance but only in tasks that require precise, high‐resolution representations (Ekstrom & Yonelinas, 2020; Yonelinas, 2013). For example, Warren et al. (2010) found that amnesic patients with MTL damage showed abnormal eye‐movement patterns in a task requiring visual search for a target among visually similar lures following a 6 s retention interval, although behavioral patterns were difficult to interpret due to near‐chance performance levels. Building on the suggestion of a hippocampal/MTL role in high‐resolution performance, Kolarik et al. (2016) characterized a densely amnesic patient with severe MTL damage as having difficulty with spatial precision in memory and navigation, while her coarse spatial ability remained largely intact. Similarly, Koen et al. (2017) reported four MTL patients (two of whom had selective hippocampal damage) who were more impaired on recognition tasks that required distinguishing between two targets that were very similar in color or location. This might suggest a deficit in high‐resolution memory, although it should be noted that the apparent impairment was not large or clear‐cut when compared with performance on the low‐resolution version of the task.

Rather than using categorical response tasks, other studies have taken the approach of measuring memory performance via continuous response tasks that require the participant to make precise judgments regarding features such as color, location, or orientation. For example, Pertzov et al. (2013) asked participants to relocate colored fractals by identifying where a probe item had originally been located or to rotate a colored bar probe until it matched the original orientation of the bar with that color from the to‐be‐remembered array. Their patient group (with limbic encephalitis mainly affecting the MTL and hippocampus) exhibited binding errors on these tasks, although their overall memory precision appeared relatively intact. Similarly, Zokaei et al. (2014, 2019) found evidence for increased binding errors in patients with MTL pathology using color–orientation or shape–location localization tasks. For example, Zokaei et al. (2019) found that a group of patients with epilepsy who had undergone anterior temporal lobectomy produced more swap errors (compared to controls) when locating colored fractals in space, at a rate that did not simply reflect item or location memory in isolation. More recently, Borders et al. (2022) employed a task in which participants used a color wheel to identify the color of one of four items that was cued by location at test. They found evidence for reduced precision (relative to controls) in a group of predominantly adult‐acquired amnesic MTL patients, some of whom were reported as having hippocampal‐selective damage.

The current study explores the generality of this evidence by examining whether a patient with highly selective, bilateral hippocampal damage (Jon) would show impairments on two different continuous response tasks designed to measure binding in working memory. To date, Jon has always displayed excellent working memory ability, in the context of severely impaired delayed recall (e.g., Allen et al., 2014; Vargha‐Khadem et al., 1997). However, our work with him has typically used tasks that require coarse, categorical recall or recognition judgments (Allen et al., 2014; Baddeley et al., 2010, 2011), leaving open the possibility that these tasks were not sufficiently sensitive to detect problems in high‐resolution working memory that might arise due to his hippocampal impairment (Yonelinas, 2013). We therefore explored whether he would produce preserved or impaired performance on continuous response tasks that take more precise measurements of recall accuracy regarding different forms of visuospatial feature binding. Specifically, this was examined in tasks measuring color–orientation binding (Experiments 1 and 3) or color–location binding (Experiments 2 and 4), using both simultaneous and sequential stimulus presentations.

## 2 EXPERIMENTS 1 AND 2: SIMULTANEOUS ENCODING

The first two experiments examined recall accuracy in two different continuous response tasks implemented under conditions of simultaneous encoding of multi‐item arrays. Experiment 1 used a color–orientation task adapted from Berry et al. (2019). In this task, participants are presented with an array of colored bars of varying orientations, followed by a test probe, with the task being to align the orientation of this bar with that seen during the encoding phase, based on its color. Experiment 2 used a color–location binding task. Here, an array of colored squares is presented, followed by a single color probe in a neutral location, with participants required to select where on screen they thought it had appeared.

Each of these tasks critically requires memory for feature binding, either between color and orientation or color and location. Jon has previously demonstrated entirely unimpaired performance on coarse categorical measures of working memory for shape–color binding (Baddeley et al., 2010) and binding between location and color or object (Allen et al., 2014). The current study asked whether he would again show intact working memory binding or if we would now see impairments when moving to a continuous response task that generates a more precise measure of accuracy.

### 2.1 Methods

#### 2.1.1 Participants

Jon is a patient with developmental amnesia resulting from selective, bilateral hippocampal damage. He was first described by Vargha‐Khadem et al. (1997) and has since been extensively reported (e.g., Allen et al., 2014; Baddeley et al., 2001; Baddeley et al., 2010; Duzel et al., 2001; Dzieciol et al., 2017; Hartley et al., 2007). He was 40 years old at time of testing for Experiments 1 and 2. There were nine control participants (all female), aged between 35 and 45 years (mean 40.22 years). Jon was tested at the Institute of Child Health, University College London, while the controls were recruited and tested at the University of Leeds. Both institutions gave ethical approval, and all participants gave informed consent.

#### 2.1.2 Design, materials, and procedure

Each of the two tasks (Experiment 1 and Experiment 2) involved six practice trials followed by 60 test trials, with memory load set at four across all trials in both tasks. These tasks were carried out within the same testing session, separated by a verbal memory task (of around 20 min duration) that is not described further here. All tasks were run using a 13” MacBook Air (resolution 1440 × 900 pixels).

**FIGURE 1:**
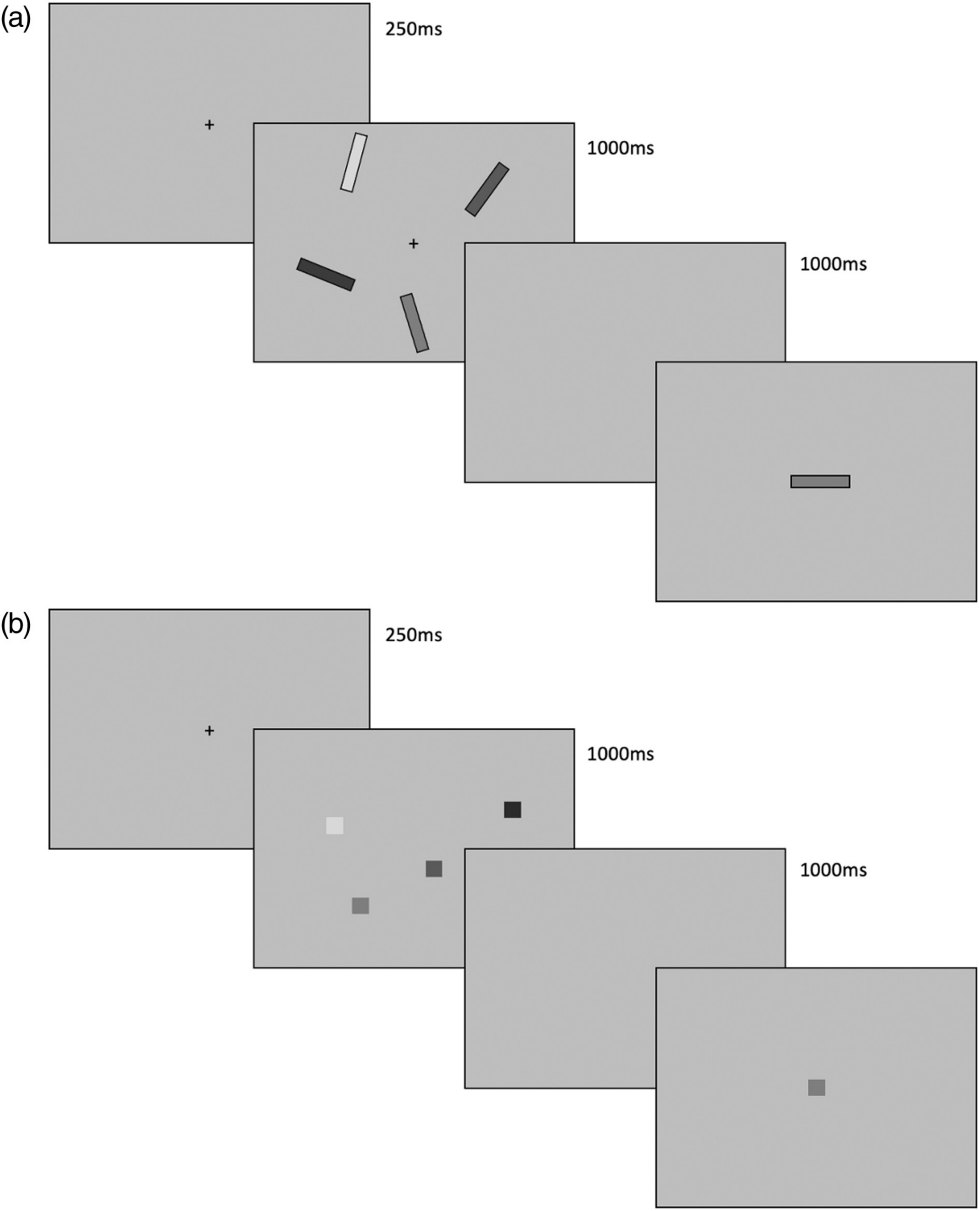
Schematic illustration of trial procedure. (a) Orientation task (Experiment 1). (b) Location task (Experiment 2). Images are not to scale, and objects in varying shades of gray represent colored stimuli

For Experiment 1, the orientation recall task was adapted from that employed in Berry et al. (Berry et al., 2019), with the task written in PsychoPy 1.84 (Peirce, 2007). Participants were presented with 4‐item arrays of colored bars each measuring 2 × 0.3 of visual angle (1.75 cm × 0.25 cm) at different orientations (see Figure 1a). The colors of the four items on each trial were randomly selected from a set of eight (blue, cyan, green, orange, pink, purple, red, and yellow) with the constraint that colors did not repeat within a trial, and orientations randomly selected so that no two bars within a given array were within 10° of one another.

Following a 250 ms fixation screen, items were presented at a randomly selected subset of eight possible locations on an invisible circle (radius of 6° visual angle) around fixation. The target array on each trial was presented for 1,000 ms, followed by a blank screen 1,000 ms retention interval. The test probe (a single bar in a horizontal orientation) was then presented at the center of the screen, in the color of one of the presented items on that trial. Participants used the left and right arrow keys to rotate the bar with the aim of matching its orientation to that encountered in the target array and pressed the enter key when they were happy with their response. A 10 s automatic timeout was built into the task.

The color–location task in Experiment 2 was run in SuperCard and was adapted from previous tasks reported in the literature (Pertzov et al., 2013; Zokaei et al., 2019). Following a 250 ms fixation screen, an array of four colored squares (1 × 1 cm each) was presented for 1,000 ms on a gray background. For each trial, these four colors were randomly drawn without repetition from a set of eight (black, blue, brown, green, purple, red, turquoise, and yellow) and were presented in randomly selected locations around the screen. Following a 1000 ms blank screen delay, one of the colored squares from the target array was re‐presented at screen center, and participants used a mouse attached to the laptop to select the location in which they thought this color had appeared. Participants again had 10 s in which to make their response.

Finally, the AB reasoning test (Baddeley, 1968) was also conducted as a proxy for IQ. Participants were provided with the list of 64 short statements (e.g., *B is preceded by A, B‐A*) on a single sheet of A4 paper and asked to indicate True or False (using a pen stroke). The total time taken to complete the set was recorded via stopwatch.

### 2.2 Results and discussion

#### 2.2.1 AB reasoning test

Independent group t tests indicated no significant difference (*p* > .05) in the number of correct responses, *t*(8) = 1.57 (Jon = 61, Control mean = 54.6, Control SD = 3.50, Max = 64) or completion time, *t*(8) = 0.89 (Jon = 5.11 min, Control mean = 4.13, Control SD = 1.04).

#### 2.2.2 Experiment 1: Color–orientation

Performance was scored and analyzed using the *Mixtur* package in R (Grange & Moore, 2022). Model‐free summary statistics (absolute angular error and the resulting estimate of precision) were first obtained via *Mixtur*. We then used this package to apply the Zhang and Luck (2008) two‐component model to the data, separating out probability of recalling the true target orientation from a uniform distribution indicating random guessing.^1^

**FIGURE 2:**
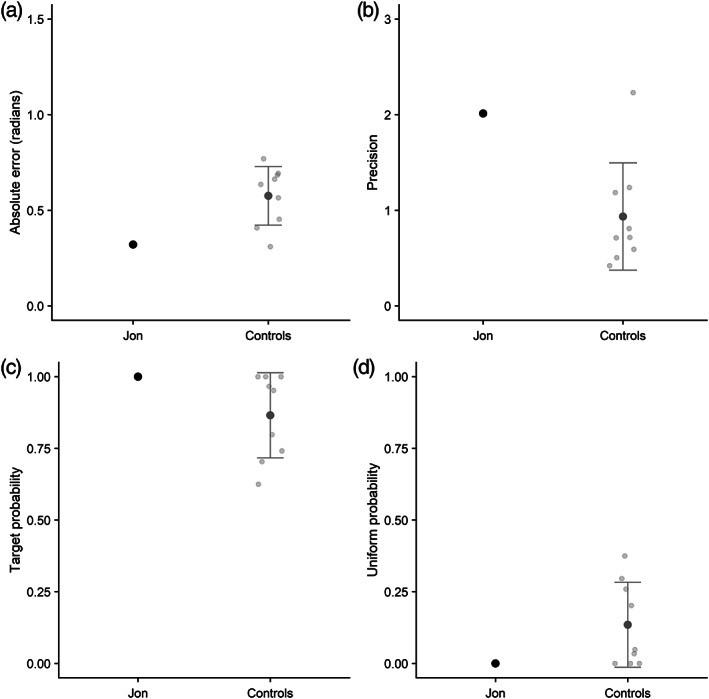
(a) Mean absolute error (radians) of recall in the simultaneous orientation task (Experiment 1) for Jon and controls; (b) precision score; (c) probability of recalling target orientation; (d) probability of a uniform guessing pattern. Individual control participants are shown in light gray, and error bar shows standard deviation (SD). Higher scores represent better performance in panels B and C, and worse performance in A and D

Mean absolute error (in radians) is reported in Figure 2a for Jon and the controls, along with an estimate of precision (Figure 2b). For both measures, Jon's performance is numerically superior to controls. However, independent group t tests (Crawford & Howell, 1998) indicated that Jon's response accuracy was not significantly different from the control group for absolute error, *t*(8) = 1.57, *p* > .05, or precision *t*(8) = 1.83, *p* > .05. For the modeling outcomes, Jon was again numerically superior to the control mean and actually achieved a probability score for target retrieval of 1.0, with 0.0 probability of a uniform guessing response, though these rates did not significantly differ from those of controls, *t*(8) = 0.87, *p* > .05.

#### 2.2.3 Experiment 2: Color–location

Performance on this task was scored in terms of both mean absolute distance (in cm) from the target location (i.e., a measure of precision of response) and a categorical correct/incorrect score. The latter score was calculated by classing as correct any response that fell within 1 cm of the target location center.

**FIGURE 3:**
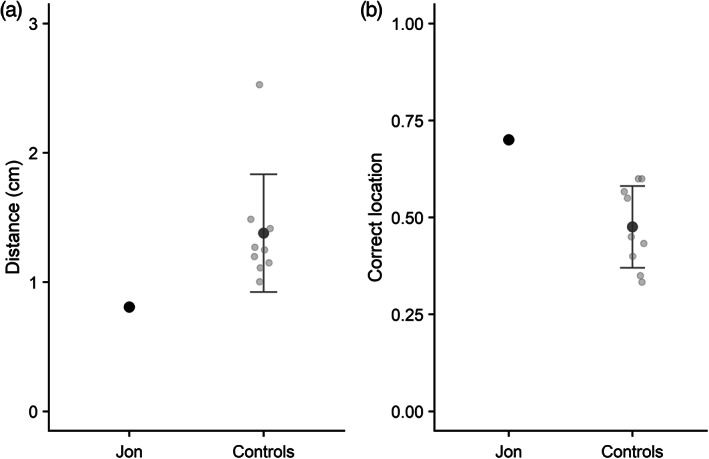
(a) Mean recall distance from target in the simultaneous location task (Experiment 2) for Jon and controls; (b) mean proportion of responses scored as categorically correct. Individual control participants are shown in light gray, and error bars show SD. Higher scores represent worse performance in panel A, and better performance in panel B

Mean distance from target location (in cm) is reported in Figure 3a for Jon and the control group. Analysis indicated that Jon and the control group did not significantly differ in their accuracy of responding, *t*(8) = 1.18, *p* > .05. This remained the case when a control participant whose response accuracy fell 2 standard deviations above the mean (see Figure 3a) was excluded from the analysis. However, Jon did perform significantly more accurately than controls when a categorical scoring measure was applied (Figure 3b), *t*(8) = 2.02, *p* < .05.

To summarize, Jon showed no evidence of impairment in response accuracy on tasks involving either color and orientation or color and location whether measured by either categorical or continuous responses. Indeed, in both cases, his mean response error was numerically superior relative to the control group, though these differences did not reach the criterion for statistical significance. Thus, his substantial bilateral hippocampal damage does not preclude this patient from responding with impressive precision in these working memory binding tasks. We also obtained categorical measures of target retrieval accuracy, either through modeling of target orientation probability versus guessing (Experiment 1) or based on whether participants responded within the correct target area (Experiment 2). Here, Jon again performed very well, achieving a perfect probability of target orientation retrieval, and a location correct score that was significantly better than that of controls.

## 3 EXPERIMENTS 3 AND 4: SEQUENTIAL ENCODING

The first two experiments established that Jon was able to respond with very high precision and accuracy in continuous response tasks measuring working memory binding. We followed this up by exploring whether the same pattern would emerge when to‐be‐remembered stimuli were presented individually in a sequence, rather than as a single‐shot display. Sequential presentation reduces the opportunity to capitalize on holistic processing of the whole array. It also introduces challenges in the requirement to encode and retain each item in a sequence, including repeated updating of working memory content, holding each item, and protecting it from retroactive interference caused by subsequent items in the sequence.

Working memory binding appears to be more vulnerable to such interference, with healthy participants showing reduced binding accuracy with serial relative to simultaneous presentation for early sequence items (Allen et al., 2006; Brown et al., 2017; Brown & Brockmole, 2010) and greater disruption from a to‐be‐ignored suffix (Ueno et al., 2011). The most recently encountered item appears to be held in a privileged state within the focus of attention and thus be more accessible, relative to early sequence items (e.g., Hitch et al., 2020), while there is also evidence of differences in MTL activation between pre‐recency and recency items (Lewis‐Peacock et al., 2012; Öztekin et al., 2010). Sequential presentation also introduces an inherent serial ordering component to the task that is absent from simultaneous presentation. Furthermore, there is evidence of a possible hippocampal contribution to serial‐order memory (Konkel et al., 2008; Long & Kahana, 2019). Thus, one or a combination of these possibilities may serve to increase the chances of finding poorer performance in the patient relative to controls. This is investigated in Experiments 3 and 4.

### 3.1 Method

#### 3.1.1 Participants

Jon was 41 years old at the time of testing. There were 11 control participants (2 females and 9 males, aged 35–46 years, mean = 41), 6 of whom also took part in Experiments 1 and 2.

#### 3.1.2 Design, materials, and procedure

These tasks were closely based on the methods implemented in Experiments 1 and 2. The key difference in Experiments 3 and 4 was that items were presented one at a time, for 250 ms per item, with a 250 ms interstimulus interval separating each display. The final item was followed by a 1,000 ms blank screen delay before the test cue was presented.

### 3.2 Results and discussion

#### 3.2.1 AB reasoning test

The control group's performance on the AB reasoning test (Baddeley, 1968) was compared against Jon's as obtained in the testing session completed the previous year (see Experiment 1 and 2). Independent group t tests indicated no significant difference (*p* > .05) in the number of correct responses, *t*(10) = 0.81 (Jon = 61, Control mean = 53.27, Control SD = 9.08; Max = 64) or completion time, *t*(10) = 0.47 (Jon = 5.11 minutes, Control mean = 4.32, Control SD = 1.58).

#### 3.2.2 Experiment 3: Color–orientation

**FIGURE 4:**
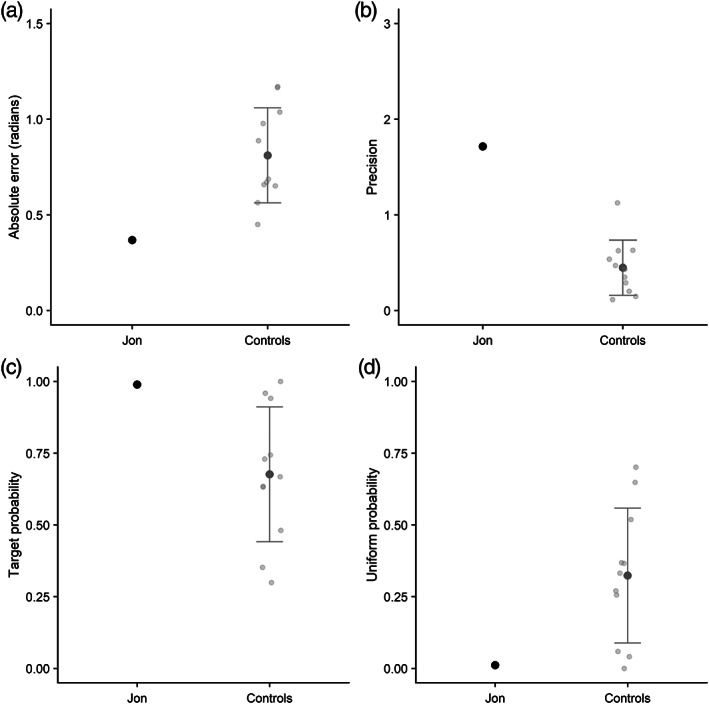
(a) Mean absolute error in the sequential orientation task (Experiment 3) for Jon and controls; (b) precision score; (c) probability of recalling target orientation; (d) probability of uniform guessing. Individual control participants are shown in light gray, and error bars show SD

Response accuracy, precision, and modeling outcomes are displayed in Figure 4. Jon's absolute error score was numerically lower (i.e., numerically better) than controls, but this difference was not significant, *t*(10) = 1.69, *p* > .05. His precision was significantly better than controls, *t*(10) = 4.23, *p* < .001. Turning to probability of target retrieval versus uniform guessing, Jon again achieved a very high probability of target retrieval (.989), though this did not significantly differ from controls, *t*(10) = 1.23, *p* > .05.

**FIGURE 5:**
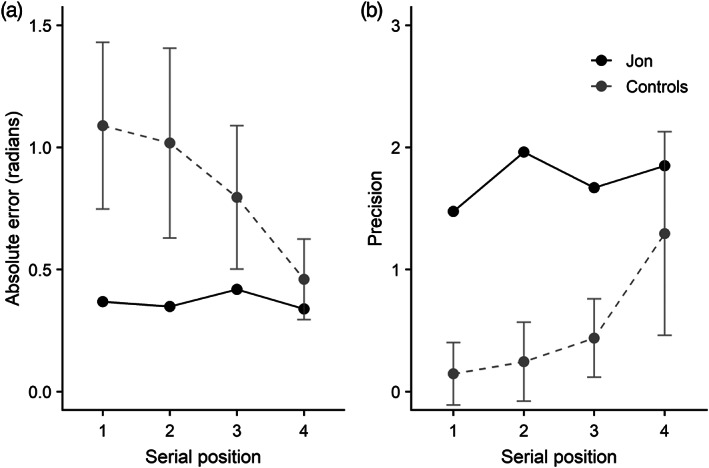
(a) Mean absolute error in the sequential orientation task (Experiment 3) for Jon and controls, by serial position; (b) precision score. Error bars show standard deviation

Response accuracy (absolute error and precision) is also reported by serial position, in Figure 5. Control participants showed notable recency effects, with error declining and precision improving toward the end of the sequence. Jon's performance demonstrates a somewhat flatter curve, likely reflective of his accurate performance overall.

#### 3.2.3 Experiment 4: Color–location

**FIGURE 6:**
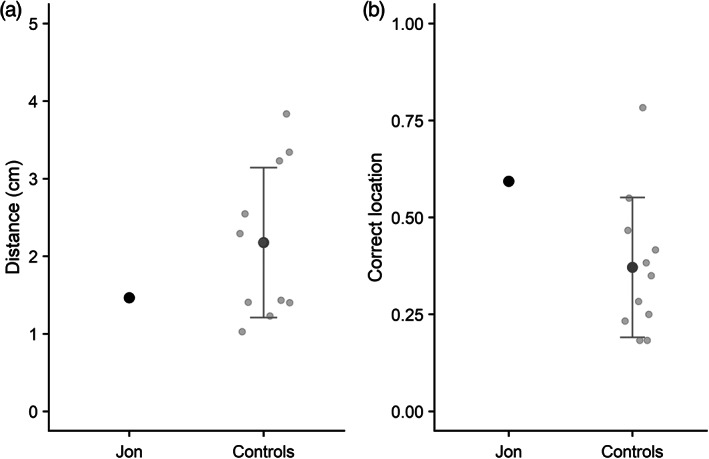
(a) Mean distance from target location in the sequential location task (Experiment 4) for Jon and controls; (b) mean proportion of responses scored as categorically correct. Individual control participants are shown in light gray, and error bars show SD

**FIGURE 7:**
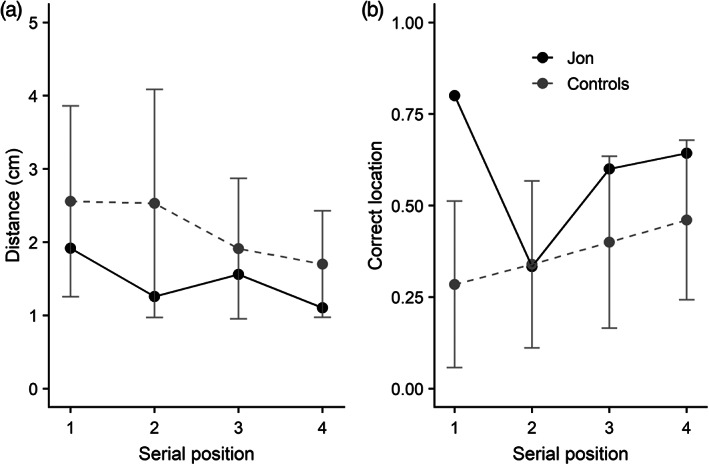
(a) Mean distance from target by serial position in the sequential location task (Experiment 4) for Jon and controls and reaction time; (b) mean proportion of responses scored as categorically correct. Error bars show SD

Mean distance from target location (in cm) is reported in Figure 6a for Jon and the control group, with proportion of categorical correct responses in Figure 6b. Jon exhibited numerically lower absolute error and numerically higher precision relative to controls, but these differences were not significant (Absolute error: *t*(10) = 0.70, *p* > .05; Precision: *t*(10) = 1.44, *p* > .05). Figure 7 presents performance by serial position for each of these measures. Both Jon and controls show some improvement in continuous response accuracy toward the end of the sequence, as measured by distance from the target. A similar pattern is apparent on the categorical outcome, though Jon does show improved accuracy for the first serial position.

Summarizing Experiments 3 and 4, performance based on sequential target presentation resulted in a pattern of results closely resembling that found in Experiments 1 and 2 using simultaneous presentation. Jon achieved relatively high accuracy scores when measured using resolution/precision of responses for both types of binding tasks, as was apparent when modeling probability of target orientation retrieval (Experiment 3) and categorical location scoring (Experiment 4).

### 3.3 Overview of relative performance across primary outcome measures

**FIGURE 8:**
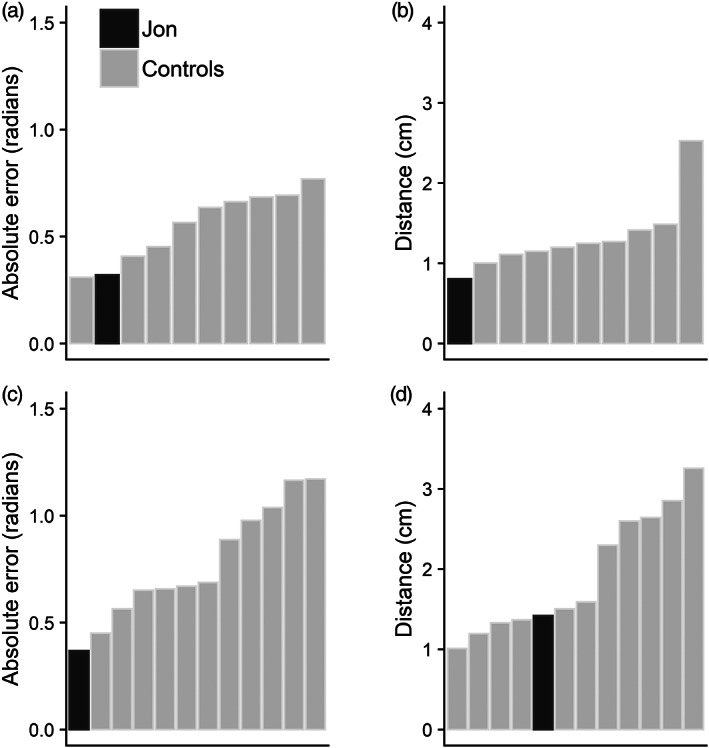
Ranking of performance from Jon (in black) and individual control participants (in light gray) on the primary outcome measure. (a). Absolute error in Experiment 1 (simultaneous orientation); (b). Distance in Experiment 2 (simultaneous location); (c). Absolute error in Experiment 3 (sequential orientation); (d). Distance in Experiment 4 (sequential location). In all panels, lower values indicate better performance

It is apparent from each of the tasks that Jon performs with relatively high accuracy across different performance measures, compared to controls. To summarize and further illustrate this, Figure 8 shows the ranking of participants on the primary outcome measure used in each task. This clearly illustrates how Jon performs with relatively high accuracy across all experiments.

## 4 GENERAL DISCUSSION

We examined the ability of Jon, a patient with relatively selective hippocampal damage, to make precise response judgments in tasks measuring binding between color and orientation (Experiments 1 and 3) or color and location (Experiments 2 and 4). Across all four experiments, and regardless of the type of binding being examined or the format of the presentation, Jon's response accuracy was high and always numerically superior to the control mean. This is further illustrated in the ranking of participants for each task, illustrating how Jon performs with relatively high accuracy across all experiments.

Previous studies examining visual working memory for features and feature binding in this patient have always used categorical tasks, requiring different forms of recognition or cued recall response in which the participant has to decide between a limited, distinct, and well‐defined set of response options (Allen et al., 2014; Baddeley et al., 2010, 2011). In these tasks, Jon always performs at least as well as control participants, if not more accurately. Based on this evidence, we have argued against a role for the hippocampus in working memory binding. It remained a possibility, however, that we were measuring an intact ability to make broad categorical judgments while failing to detect an underlying impairment on tasks requiring a more precise response, in keeping with the suggestion of Yonelinas (2013) that the hippocampus supports high‐resolution cognition.

Here, we show for the first time that Jon is in fact able to make very precise responses in different types of working memory binding tasks. When tasked with retrieving color–orientation or color–location binding information, Jon produced a relatively low absolute error rate and high precision scores (where these were available, in Experiments 1 and 3). Modeling of responses in Experiments 1 and 3 also indicated an extremely high probability of retrieving the target orientation in response to a color probe, while Experiments 2 and 4 also demonstrated that Jon was very accurate, relative to controls, in selecting the precise categorical location in which the probe color had originally appeared.

Not only did we find convergent findings across binding tasks but also across presentation formats. Participants were in general somewhat less accurate with sequential compared to simultaneous presentation and showed reduced error and improved precision on trials where the probed item was drawn from the end of the sequence. These overall patterns are in line with findings from studies using categorical (e.g., Allen et al., 2006, 2014; Allen et al., 2017; A. L. Atkinson et al., 2018; Hu et al., 2014) and continuous response tasks (e.g., Gorgoraptis et al., 2011), and fit with the claim that bound representations are fragile and vulnerable to interference caused by subsequently encountered stimuli (Allen et al., 2006; Ueno et al., 2011). However, there was no sign that Jon has relatively greater difficulty (compared to controls) when stimuli are presented sequentially (Experiments 3 and 4) rather than simultaneously (Experiments 1 and 2). Thus, Jon does not show any evidence of struggling with working memory binding updating or loss of early sequence items due either to retroactive interference or to being displaced from the focus of attention. Indeed, his profile of performance across serial positions, while obviously being noisier than the control mean, does not indicate relative impairment at any list position. These findings would also indicate, at least in Jon, the independence of serial‐order memory from the hippocampus (cf. Long & Kahana, 2019), though the current tasks implemented single‐item probe measures and so did not explicitly require serial ordering. Previous working memory studies with Jon have shown good performance on serial order recall tasks (Baddeley et al., 2010, 2011), but it may be worthwhile implementing high‐resolution measures in that context.

Rather than showing any sign of impairment in retrieval accuracy, it is apparent when considering Jon's performance alongside that of controls that he performs very well across various measures of recall accuracy (e.g., for retrieval error, see Figure 7). This is not unusual for this individual; in previous experimental explorations of visuospatial working memory (Allen et al., 2014; Baddeley et al., 2010, 2011), Jon has often produced response accuracy levels that are at least numerically higher than those of control participants. For example, Baddeley et al. (2010) found that Jon was at least numerically superior to the control mean on all three recognition‐based measures of shape–color binding (examining unitized, spatially separated, and cross‐modal feature combinations). Similarly, in measures of color–location memory (Allen et al., 2014; Study 1), Jon's recognition accuracy matched the highest achieving control participant, while his reconstruction performance was superior to 6 of the 7 controls (though he was clearly impaired in delayed tests assessing the same material). The present study replicates these patterns from categorical tasks using different continuous response tasks. Aside from tasks such as recall from episodic long‐term memory (Baddeley et al., 2001), Jon is an intelligent individual and approaches tasks in a careful and motivated manner, as also illustrated in the present study by his relative performance on the AB reasoning task. More broadly, Baddeley et al. (2011) found that Jon was more accurate than most controls on Raven's matrices, digit and Corsi span, and at least some of the complex working memory span tasks they administered. His performance IQ (using the Wechsler revised test) has been found to be in the high range by Baddeley et al. (2001). Thus, for measures that do not index his areas of impairment, he typically always appears as a relatively high‐functioning individual. To contextualize this profile of performance, although other patients with developmental amnesia have not typically been assessed on the same range of experimental measures as Jon, they typically exhibit a range of performance on working memory tasks that align with that produced by healthy controls, with Jon within this range (e.g., Dzieciol et al., 2017).

The outcomes in this study contrast with findings from MTL patients suggesting binding deficits using precision‐based continuous response tasks (e.g., Pertzov et al., 2013; Zokaei et al., 2019). Those studies report an increased tendency from patients for “swap” errors involving retrieval of nontargets, with the suggestion that binding deficits are apparent when measured using such tasks. While our analysis focused on absolute error, precision, and target retrieval probability for Experiments 1 and 3, additional modeling of the data from those experiments (reported in Supplementary Information) also indicated that Jon was not more likely than controls to make such swap errors and retrieve nontargets; indeed, this modeling indicated an extremely low probability of making such errors, which was numerically reduced compared to controls.

The present findings also contrast with those of a recent study indicating relatively reduced precision on a color–location binding task in a group of nine amnesic patients (Borders et al., 2022). However, their predominantly adult‐onset sample was relatively heterogeneous with a mix of unilateral and bilateral patients and 3/9 patients having hippocampal‐selective damage as indicated by MRI. This patient group also demonstrated a substantial performance range; although a subset of the sample exhibited a large reduction in the proportion of responses categorized as being close to the target item, at least the same number of patients achieved performance levels that were approximately equivalent to the control group mean. This variability in patient profile and performance across the amnesic group, along with the different paradigms used, makes drawing clear links between this and our study quite challenging. We would suggest that one useful step forward in resolving such apparent conflicting findings might be for research groups to compare relevant patients on a shared suite of tasks, perhaps adopting an approach of adversarial collaboration (Cowan et al., 2020).

This would also help in confirming whether any apparent variability in patterns of deficit might be attributed to methodological differences between studies. For example, Borders et al. (2022) required precise memory for color (cued via location), whereas the current study required precise memory for orientation or location when memory was cued via color. Presentation duration also slightly varied across methods; Borders et al. (2022) allowed the equivalent of 100 ms per item in their simultaneous four‐item displays, compared with 250 ms per item in simultaneous and sequential displays in the current study. Although such differences are unlikely to offer a root cause for the apparent disparate findings observed, it would be useful to establish how patient groups fare using uniform sets of procedures.

On a similar note, existing studies in this area have sometimes found clearer deficits at slightly extended intervals (i.e., 4–5 s or more, compared to shorter delays of around 1 s) in either categorical (e.g., Braun et al., 2008; Jeneson et al., 2012; Olson et al., 2006) or continuous response tasks (Zokaei et al., 2019). The present study limited the retention interval to 1 s, in line with the interval durations that are often examined in healthy individuals (Hitch et al., 2020). A previous examination of Jon's memory in response tasks over time courses of up to 10 s yielded no evidence of any deficit across this time span, in contrast to impairment in a surprise follow‐up test administered several minutes later (Allen et al., 2014). However, that study implemented categorical response tasks that were not necessarily able to detect any changes in memory resolution/precision. Thus, a further possibility for future work to explore is that memory precision following hippocampal damage might be intact at short delays but abnormally decline over the time course of several seconds. However, longer delays may increase reliance on the possible contribution of LTM. Indeed, it may prove insightful to systematically control and manipulate contributions from LTM to working memory precision. If patients such as Jon have to rely solely on otherwise intact working memory in the absence of long‐term memory influence, this should be detectable in the form of differential profiles relative to controls in tasks that vary in the extent to which LTM can enhance or inhibit performance.

As noted by Squire and Wixted (2011), once a threshold of hippocampal atrophy is reached (perhaps around 40%), the hippocampus can effectively become non‐functional. It remains to be seen if this might apply in Jon's case, in the context of working memory performance. It is established that Jon has around 50% bilateral hippocampal volume reduction (Dzieciol et al., 2017; Gadian et al., 2000), severe enough to render his delayed recall ability in episodic memory tasks to be nonfunctional, in contrast to his apparently intact working memory. On the one hand, Maguire et al. (2001); Maguire et al. (2010) suggested that bilateral activation in his residual hippocampal tissue, along with hippocampal–cortical connectivity that differed from healthy controls, may enable some functionality in his autobiographical memory and future thinking ability. However, a subsequent fMRI study by Mullally et al. (2014) indicated that Jon engaged several brain regions similar to controls when performing a scene construction task but (unlike controls) exhibited no activity changes in his remnant hippocampal tissue. Moreover, there is no current evidence that an analogous combination of residual hippocampal activity and altered cortical interactivity might also serve to support working memory.

It is of course also important to note the early developmental onset of Jon's impairment. Processes of neural compensation and reorganization due to early plasticity might serve to support independence from the hippocampus at least for certain kinds of memory operations in patients such as Jon with developmentally acquired hippocampal atrophy and amnesia. Indeed, Vargha‐Khadem et al. (2003) found that performance on some verbal tasks drawn from established neuropsychological tests (e.g., immediate story recall from the Wechsler Memory Scale (Wechsler, 1945, 1997)) was more likely to be spared in early compared to later acquired developmental amnesia. It would be important to establish whether the same kind of pattern is apparent across a range of additional measures, including those implemented in the present study, and compare it with patients who have experienced hippocampal damage later in life. Along these lines, Finke et al. (2013) (see also Braun et al., 2008) examined memory performance for color–location associations assessed over 5 s delays in epilepsy patients with unilateral hippocampal damage following right MTL resection. They found an associative impairment in patients whose epilepsy had been caused by a benign brain tumor but not in patients who had focal hippocampal sclerosis. Finke et al. argued that intact memory function in the latter group was supported by recruitment of the contralateral hippocampus and a network of distributed neocortical regions and suggested this may reflect the possible developmental onset of hippocampal pathology in this group. However, given the group studied by Finke et al. consisted of epilepsy patients with unilateral (rather than bilateral) damage, drawing firm conclusions in terms of hippocampal specificity is not straightforward.

In Jon's case, his substantial focal hippocampal damage is bilateral, symmetrical, and severe in nature. We would suggest that cortical plasticity cannot compensate for hippocampal specificity, and second that working memory is not hippocampal‐specific. Instead, one possibility is that the perirhinal, entorhinal, and parahippocampal cortices subserve the binding of information in the short term (see Miyashita, 2019). These areas may encode and bind information and act as gateways into the hippocampus for pattern consolidation and subsequent retrieval from episodic long‐term memory. In the presence of early acquired bilateral hippocampal damage, this role of the perirhinal, entorhinal, and parahippocampal cortices may become exaggerated because of plastic changes, depending on the degree of hippocampal damage and the integrity of the cortices (Chareyron et al., 2021).

Our findings demonstrate that high‐resolution working memory binding *can be* functionally independent of the hippocampus and unaffected by substantial and selective early hippocampal atrophy. This pattern of intact performance is apparent across multiple measures of performance on two different feature binding response tasks, using either simultaneous or sequential presentation. It remains for future work to establish the boundary conditions for any hippocampal‐critical working memory functioning, in terms of either task features (such as memory load, duration, presentation format, or response mode) or individual differences in patient profile (e.g., lesion selectivity or age of onset).

## Supporting information


Table S1

## Data Availability

The data presented in this study are available on request from the corresponding author.

## Footnotes

^1^
We report the two‐component model, rather than the three‐component model of Bays et al. (Bays et al., 2009), for two reasons. First, simulations by Grange and Moore (2022) indicate that the two‐component model can provide good parameter outcomes using 50 trials. Second, given Jon's strong performance overall, and very high probability of retrieving the target, it was extremely unlikely for him to produce inflated swap errors as measured by the three‐component approach. Nevertheless, we report the three‐component outcomes in Supplementary Information for Experiments 1 and 3.
