# Supplementary material for: Intact high‐resolution working memory binding in a patient with developmental amnesia and selective hippocampal damage
Source: Hippocampus. 2022 Jun 23;32(8):597–609. doi: 10.1002/hipo.23452 (PMC9542612; doi:10.1002/hipo.23452)
Supplement: Supplementary file 1 — Table S1 [file HIPO-32-597-s001.docx]

Supplementary materials


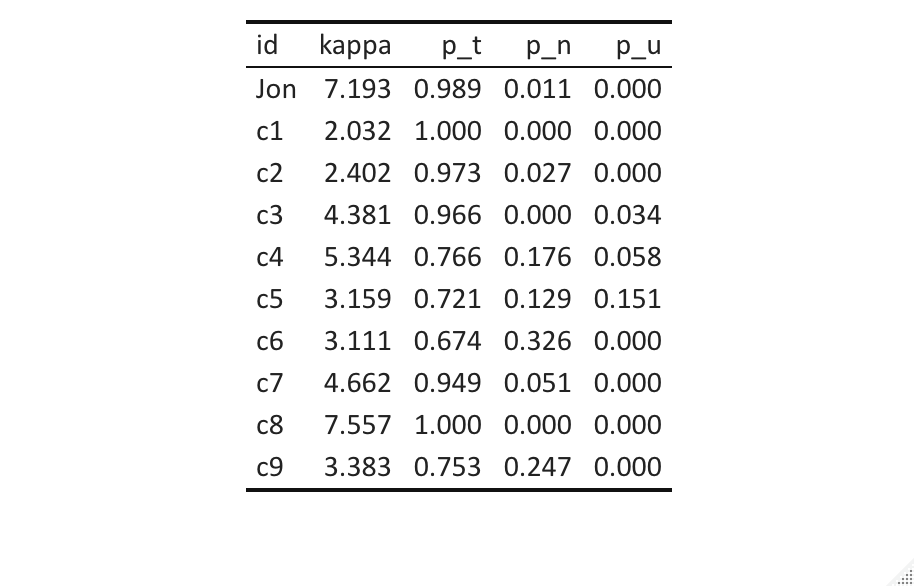
Table S1. Three-component model outcomes derived using *Mixtur* [45] for Jon and control participants from Experiment 1 (simultaneous presentation). p_t indicates target probability, p_n is non-target probability, and p_u the uniform response distribution.


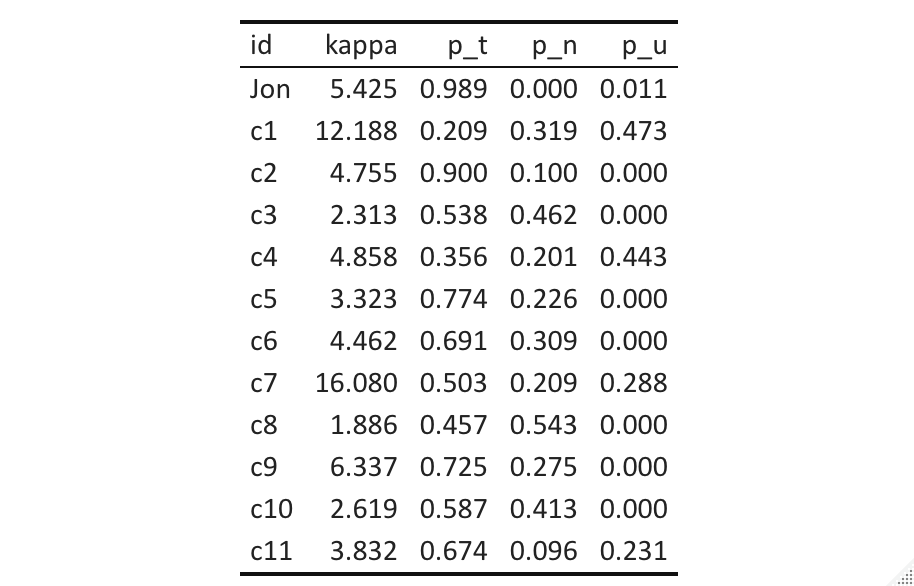
Table S2. Three-component model outcomes (derived using *Mixtur*) for Jon and control participants from Experiment 2 (sequential presentation). p_t indicates target probability, p_n is non-target probability, and p_u the uniform response distribution.
